# Chronic pain, perceived stress, and cellular aging: an exploratory study

**DOI:** 10.1186/1744-8069-8-12

**Published:** 2012-02-12

**Authors:** Kimberly T Sibille, Taimour Langaee, Ben Burkley, Yan Gong, Toni L Glover, Chris King, Joseph L Riley, Christiaan Leeuwenburgh, Roland Staud, Laurence A Bradley, Roger B Fillingim

**Affiliations:** 1 College of Dentistry, University of Florida, P.O. Box 103628, Gainesville, FL 32610-3628, USA; 2 College of Pharmacy and Center for Pharmacogenomics, University of Florida, Gainesville, USA; 3 College of Dentistry and College of Nursing, University of Florida, Gainesville, USA; 4 College of Medicine, University of Florida, Gainesville, USA; 5 Division of Clinical Immunology and Rheumatology, University of Alabama at Birmingham, Birmingham, USA; 6 College of Dentistry, University of Florida and North Florida/South Georgia Veterans Health System, Gainesville, USA

**Keywords:** Chronic pain, Perceived stress, Knee osteoarthritis, Telomere length, Cellular aging

## Abstract

**Background:**

Chronic pain conditions are characterized by significant individual variability complicating the identification of pathophysiological markers. Leukocyte telomere length (TL), a measure of cellular aging, is associated with age-related disease onset, psychosocial stress, and health-related functional decline. Psychosocial stress has been associated with the onset of chronic pain and chronic pain is experienced as a physical and psychosocial stressor. However, the utility of TL as a biological marker reflecting the burden of chronic pain and psychosocial stress has not yet been explored.

**Findings:**

The relationship between chronic pain, stress, and TL was analyzed in 36 ethnically diverse, older adults, half of whom reported no chronic pain and the other half had chronic knee osteoarthritis (OA) pain. Subjects completed a physical exam, radiographs, health history, and psychosocial questionnaires. Blood samples were collected and TL was measured by quantitative polymerase chain reaction (qPCR). Four groups were identified characterized by pain status and the Perceived Stress Scale scores: 1) no pain/low stress, 2) no pain/high stress, chronic pain/low stress, and 4) chronic pain/high stress. TL differed between the pain/stress groups (*p *= 0.01), controlling for relevant covariates. Specifically, the chronic pain/high stress group had significantly shorter TL compared to the no pain/low stress group. Age was negatively correlated with TL, particularly in the chronic pain/high stress group (*p *= 0.03).

**Conclusions:**

Although preliminary in nature and based on a modest sample size, these findings indicate that cellular aging may be more pronounced in older adults experiencing high levels of perceived stress and chronic pain.

## Findings

A recent Institute of Medicine report documents the public health consequences of chronic pain in America with estimates of 116 million adults affected and costs of $635 billion annually [1]. One of the challenges illuminated in the report is the difficulty in identifying specific pathophysiological targets due to significant variability in the experience of chronic pain. Consequently, biological markers reflecting the physiological burden of chronic pain on an individual system would offer significant clinical and scientific utility.

Leukocyte telomere length (TL) is a measure of cellular aging and is associated with age-related disease onset, chronic health conditions, psychosocial stress, and mortality [2-5]. Importantly, recent findings indicate a direct relationship between telomeres and mitochondria, connecting for the first time two major theories of aging [6]. Telomeres are the protective structures at the end of chromosomes comprised of DNA repeat sequences which are reinforced by telomerase activity [7]. Although buffered by telomerase activity, TL decreases over time with cell replication. However, the rate of attrition appears to be influenced by numerous factors including the "biochemical environment" such as oxidative stress, inflammation, and stress hormones [8,9]. Chronic pain represents a physiological stressor often associated with a cascade of negative psychosocial factors [10]. The impact of chronic pain and related psychosocial stress on cellular aging has not yet been explored.

The relationship of chronic pain associated with knee osteoarthritis (OA) and perceived stress on telomere length provides a model for studying cellular aging and chronic pain. We hypothesized that a cumulative effect of "system burden" would be observed such that individuals endorsing chronic pain and high stress would have shorter telomeres than individuals with either chronic pain or high stress, or pain-free individuals with low stress; who would have the longest telomeres of the four groups.

## Methods

Thirty six subjects, between the ages of 47 and 75, were included in the analysis. Eighteen subjects presented with current knee pain, endorsed experiencing chronic pain, were confirmed to have radiographic knee osteoarthritic changes [11], and were without rheumatoid arthritis, heart disease or uncontrolled medical conditions including high blood pressure, diabetes, and gout. The no pain group was comprised of 18 individuals presenting without chronic knee pain or medical comorbidities. The research was conducted on the Clinical Research Unit at the University of Florida (UF). The protocol and procedures were approved by UF's IRB and informed consent was obtained for each participant. Methods described are limited to those relevant to the current analyses.

Subjects completed a health history questionnaire, the Graded Chronic Pain Scale (GCPS) [12], the Perceived Stress Scale, 10 item (PSS) [13], the Center for Epidemiologic Studies Depression scale (CES-D) [14], and a physical exam including knee radiographs. Additionally, duration of knee pain in months was collected. Baseline blood samples were collected and peripheral blood mononuclear cells (PBMCs) were isolated to assess TL. Genomic DNA was extracted from lymphocytes in whole blood using a commercially available kit (Qiagen Flexigene DNA Kit, Qiagen, Valencia, CA). DNA samples were quantified, normalized and plated in 96-well plates. Relative telomere length was measured by quantitative real-time polymerase chain reaction (qPCR). Each qPCR experiment contained telomere primers and β2-globin (control gene) primers [15].

Telomeres and a single copy gene (β2-globin) were amplified in all the samples in triplicate on each plate. The ΔΔCt method was performed using SDS V.2.1 software from Applied Biosystems. Ct values from each sample were then used to calculate the ratio of telomere to single copy gene (T/S) values using the formula (2 C_t_^telomere^/2 C_t_^β2-globin^)^-1^. Relative T/S values were calculated by the formula 2 ^-ΔΔCt. ^Using this formula, we determined the TL of each sample relative to the mean T/S for all samples [15].

Independent sample t-tests, ANOVA, and Chi Square or Fisher's Exact tests were implemented for between group comparisons of demographic variables as appropriate. Due to the limited sample size, covariates were selected based on the following criteria: 1) previous association of the measure with TL, 2) association with TL in the current study, 3) not significantly correlated with other covariates, and 4) relevance to the study design. Based on previous findings, potential covariates considered included age, sex, waist/hip ratio (WHR), BMI, annual income, education, exercise frequency, smoking status, and depression. A median PSS split was used to categorize low stress (< 15) and high stress (≥ 15) as previously reported [16]. Four groups were developed based on pain status, no pain or chronic pain, and PSS total scores and were categorized as no pain/low stress (NPLS); no pain/high stress (NPHS); chronic pain/low stress (CPLS); and chronic pain/high stress (CPHS). General Linear Models (GLM) were implemented to analyze TL data. All analyses were completed with IBM SPSS Statistics 19.

## Results

**Figure 1:**
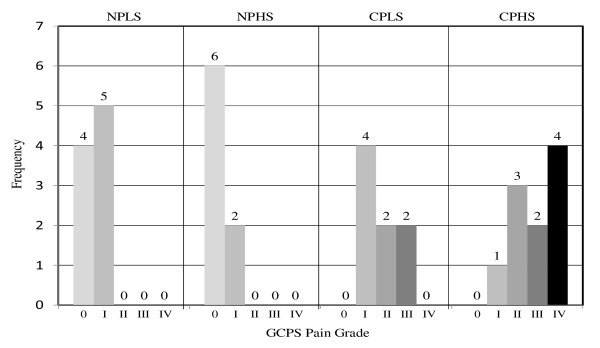
**GCPS Pain Grade by Pain/Stress Group**. NPLS - no pain/low stress; NPHS - no pain/high stress; CPLS - chronic pain/low stress; CPHS - chronic pain/high stress GCPS - Graded Chronic Pain Scale Grade Classification [12]: 0 - Pain Free; I - Low disability, low intensity; II - Low disability, high intensity; III - High disability, moderately limiting; IV - High disability, severely limiting.

**Figure 2:**
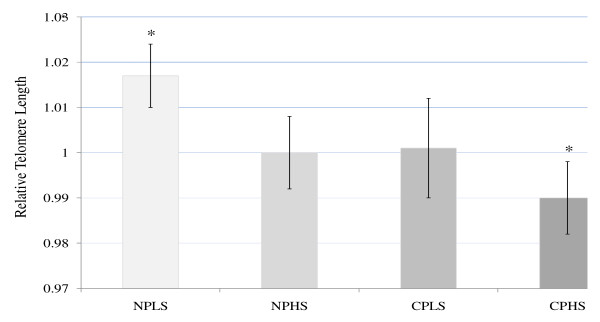
Relative Telomere Length (Estimated Mean) by Pain/Stress Groups after Adjustments for Age, Race, Waist-Hip Ratio (WHR), and WHR*Group Interaction. Mean and Standard Error: NPLS - no pain/low stress (1.017 ± .007) NPHS - no pain/high stress (1.000 ± .008). CPLS - chronic pain/low stress (1.001 ± .011). CPHS - chronic pain/high stress (.990 ± .008). Covariate values in the model: WHR, race, and age. *Significant at *p *< 0.05, Least Significant Difference (LSD).

Mean duration of knee pain for the chronic pain group was 94.8 ± 93 months (n = 17). TL did not differ between the no pain and chronic pain groups (all *p*'s > 0.10) but did differ between the low stress and high stress groups (*p *= 0.02) with covariates in the model. Descriptive data by pain/stress group are presented in Table 1 and Figure 1. TL was correlated with age (r = -0.34, *p *= 0.04) and WHR (r = -0.33, *p *= 0.05). Further analysis of age and TL stratified by pain/stress groups revealed a significant correlation only in the CPHS group (r = -0.69, *p *= 0.03) with non-significant associations in the other three groups (r = -.05/NPLS; -.24/NPHS; -.26/CPLS, *p *> 0.05). Based on previously described criteria, covariates retained in the multivariate model were age, WHR, and race. Additionally, as a result of a significant interaction between WHR and the pain/stress groups an interaction term was also included in the group comparison analyses. Incorporating age, race, WHR, and WHR*group interaction into the model, TL differed across the pain/stress groups, F (3, 26) = 4.33, *p *= 0.01, η^2 ^= 0.333 (Figure 2). Pairwise comparisons using Least Significant Difference (LSD) indicated NPLS and CPHS group differences (*p *= 0.02).

**Table 1:** Descriptor Variables by Pain and Stress Groups (Means, Standard Deviations, and Percentiles)

Group	No Pain	No Pain	Chronic Pain	Chronic Pain
	Low Stress	High Stress	Low Stress	High Stress
	(n = 10)	(n = 8)	(n = 8)	(n = 10)
Age	57.3 ± 6.0	64.2 ± 5.7	57.8 ± 8.4	57.5 ± 7.1

Sex^ŧ^	80% W	75% W	50% W	90% W
	20% M	25% M	50% M	10% M

Race^ŧ^	10% AA	12.5% AA	37.5% AA	50% AA
	90% NHW	87.5% NHW	62.5% NHW	50% NHW

WH Ratio	0.82 ± 0.098	0.82 ± 0.104	0.91 ± 0.084	0.81 ± 0.080

PSS*	9.7 ± 2.8	19.2 ± 2.9	9.5 ± 4.3	20.9 ± 6.0

CES-D*	4.0 ± 3.9	7.4 ± 4.8	7.6 ± 4.0	14.9 ± 7.8

*AA *African American; *CES-D *Center for Epidemiological Studies Depression Scale; *M *men; *NHW *non-Hispanic white; *PSS *Perceived Stress Scale; *W *women

*Significant at *p *< 0.005

^ŧ^Though not significant, proportions differ

## Discussion

This is the first study to assess the relationship of chronic pain and perceived stress with cellular aging. TL has been identified as a cumulative marker of psychosocial stress and chronic disease states [2,17,18]. Findings from the current investigation indicate that individuals endorsing a cumulative and combined burden of chronic pain and high stress have shorter telomeres than individuals without chronic pain and endorsing low levels of stress.

Prior studies have indicated a small and negative correlation between age and TL length [2,19,20]. We also found a small, negative correlation between age and TL across groups which upon further analysis was limited to a strong and significant correlation in the chronic pain/high stress (CPHS) group. These findings suggest that age-related changes in TL may be more pronounced when combined with pain chronicity and high levels of stress and align with 1) patterns previously reported regarding shorter TL in an older group of adults with anxiety disorders compared to similar aged controls [21] and 2) literature indicating stress accelerating the effects of aging and immune vulnerability in older adults [22].

Finally, TL has been negatively associated with perceived stress in 20-50 year old women [2] but not in a group of 50-70 year old men and women [16]. Adjusting for age, race, and WHR, data from the current study indicate that high levels of stress are negatively associated with TL regardless of pain status. Importantly, the association of stress with TL may be modulated by additional factors. For example, Puterman and colleagues [23] reported that exercising at a physical activity level recommended by the Centers for Disease Control appeared to protect women reporting higher levels of perceived stress from TL shortening. In contrast, our study suggests that chronic pain may strengthen the association of stress with reduced TL, because, the shortest telomeres were observed in the presence of chronic pain and high stress.

Although the sample size in the current study is rather small, it is within the range of prior published TL studies of preliminary findings [17,24,25] and in other studies analyzing the lowest and highest tertiles or quartiles of a sample [2,16,23]. More detailed information regarding knee pain duration (beyond 6 months), intensity, and persistence is necessary to better understand pain characteristics that may contribute to telomere shortening. Also, assessment of chronicity and recurrence of mood disorders, in addition to current depressive symptoms would be helpful. These limitations notwithstanding, findings encourage further exploration of the potential utility of TL in improving our understanding of individual differences in the biological consequences of chronic pain conditions. Future studies with a larger sample size will allow for more stringent adjustments for multiple comparisons and further investigation of potentially relevant covariates. Additionally, determining the predictive utility of TL in longitudinal designs would have significant clinical and research value.

## Conclusions

Unlike other disease states with documented pathophysiological markers, the biological interface of the experience of chronic pain conditions has been more difficult to measure. Though exploratory, the current findings provide preliminary evidence that chronic pain and psychosocial stress may impose a "burden on the system," accelerating cellular aging.

## Abbreviations

CES-D: Center for Epidemiological Studies Depression Scale; CPHS: Chronic pain/high stress; CPLS: Chronic pain/low stress; GCPS: Graded Chronic Pain Scale; NPHS: No pain/high stress; NPLS: No pain/low stress; OA: Osteoarthritis; PBMC: Peripheral blood mononuclear cells; PSS: Perceived Stress Scale; TL: Telomere length; T/S: Telomere to single copy gene; UF: University of Florida; WHR: Waist hip ratio.

## Competing interests

Roger Fillingim, Ph.D. is a stockholder in Algynomics.

## Authors' contributions

KTS was responsible for study conception and design, data collection, analysis and interpretation of results, and drafting of the manuscript. TL was involved in study conception and design, telomere analysis, and manuscript development and revision. BB was involved in study conception and design, blood sample processing and telomere analysis, and manuscript development and revision. YG contributed to study conception and design, assisted with analysis and interpretation of results, and manuscript development and revision. TLG assisted with parent study conception and design, involved in data collection, and manuscript critique and revision. CK assisted with parent study conception and design, involved in data collection, and manuscript critique and revision. JR contributed to study conception and design, assisted with analysis and interpretation of results, and manuscript critique and revision. CL was involved in study conception and design, assisted with biomarker protocol, and manuscript critique and revision. RS was involved in parent study conception and design, medical oversight of study participants, and manuscript critique and revision. LAB was responsible for parent study conception, design, and acquisition of funding, and manuscript critique and revision. RBF was responsible for current study and parent study conception, design, and acquisition of funding; oversight of data collection, analysis, and interpretation of results; and drafting of the manuscript. All authors reviewed and approved the final manuscript.
